# Exploring neural activity changes during motor imagery-based brain-computer interface training with robotic hand for upper limb rehabilitation in ischemic stroke patients: a pilot study

**DOI:** 10.3389/fnhum.2025.1626000

**Published:** 2025-10-24

**Authors:** Yiqing Lu, Weiwei Yang, Song Wu, Yicheng Li, Jinhu Wei, Ming Li, Yongcheng Li, Yaping Huai

**Affiliations:** ^1^Department of Rehabilitation Medicine, Shenzhen Longhua District Central Hospital, Shenzhen, Guangdong, China; ^2^Shenzhen Longhua District Rehabilitation Medical Equipment Development and Transformation Joint Key Laboratory, Shenzhen, Guangdong, China; ^3^CAS Key Laboratory of Human-Machine Intelligence-Synergy Systems, Shenzhen Institutes of Advanced Technology (SIAT), Chinese Academy of Sciences (CAS), Shenzhen, Guangdong, China

**Keywords:** brain-computer interface, EEG, ischemic stroke, motor imagery, robotic hand, upper limb rehabilitation

## Abstract

**Objective:**

This pilot study aimed to evaluate the feasibility and tolerability of motor imagery (MI)-based brain-computer interface (BCI) training with robotic hand assistance for upper limb rehabilitation, and to explore preliminary neural markers in ischemic stroke patients.

**Methods:**

Three post-stroke participants performed MI tasks combined with exoskeleton-assisted movements to facilitate rehabilitation training. Electroencephalography (EEG) signals were recorded to assess the neural correlates of MI. Functional outcomes were evaluated using standard assessment tools.

**Results:**

Our results demonstrated significant improvements in motor function across all participants. Additionally, EEG analysis revealed event-related desynchronization (ERD) in the high-alpha band power at motor cortex locations, with individual differences in both the frequency and power of neural activity. However, no significant trends in neural activity were observed across the training sessions.

**Conclusion:**

These findings suggest that MI-based BCI training, combined with robotic assistance, offer a promising approach for enhancing upper limb function in ischemic stroke patients.

## 1 Introduction

Upper limb motor dysfunction following stroke is one of the most common and disabling symptoms, significantly impacting the quality of life of patients. Restoring upper limb function is crucial for improving the independence of patients in daily activities and facilitating their social participation. Traditional rehabilitation methods, including physical and occupational therapy, can improve motor function to some extent; however, there is still room for improvement in terms of rehabilitation effectiveness and sustainability. Therefore, accelerating the recovery of upper limb motor function has become a major challenge in both clinical medicine and neuroscience research.

In recent years, brain-computer interface (BCI) technology has brought new hope for stroke rehabilitation (Li et al., 2025; Liu et al., 2025). Among the various approaches, BCI systems based on MI combined with robotic hand applications have emerged as a promising method (Bhagat et al., 2020; Kawakami et al., 2016; Lin et al., 2022; Tsuchimoto et al., 2019). In this approach, patients activate the motor areas of the cortex through MI, generating neural signals that are decoded by the BCI system to control a robotic hand for upper limb training (Frolov et al., 2017). This type of training not only provides patients with more intuitive feedback but also allows them to establish a link between neural activity and physical movement through the robotic hand. Consequently, it enhances cortical plasticity, promotes broader neural network reorganization, and accelerates motor function recovery (Bai et al., 2020; Baniqued et al., 2021). The integration of the robotic hand provides direct feedback during MI training, enabling patients to perceive the movement process and gradually regain upper limb motor abilities through continuous training.

The dynamic changes in electroencephalography (EEG) signals during MI, particularly the phenomena of event-related desynchronization (ERD) and event-related synchronization (ERS) (Pfurtscheller and Lopes da Silva, 1999), are crucial indicators in the study of MI and motor control (Jeon et al., 2011). ERD and ERS are particularly relevant in MI tasks, where the brain exhibits distinct oscillatory patterns associated with imagined movement. During MI, ERD typically occurs in the alpha and beta frequency bands over sensorimotor regions, reflecting the suppression of motor cortex activity as the brain prepares for movement, even without apparent motor execution (Yu et al., 2022). ERD has been studied as an indicator of motor cortex activation (Chen et al., 2021; Kaiser et al., 2014). In contrast, ERS is commonly observed after the cessation of movement or during the recovery phase (Wang et al., 2023), reflecting the re-engagement and reorganization of the motor cortex. In the context of stroke rehabilitation, we hypothesize that ERD and ERS patterns can serve as biomarkers of cortical reorganization, indicating the brain ability to adapt and compensate for damage to motor areas.

The changes in ERD and ERS not only provide the neurophysiological foundation for the MI process but also serve as key signals for decoding in BCI systems (Vidaurre et al., 2021). Modulating these oscillatory patterns through BCI training, such as with robotic exoskeletons, holds potential for promoting functional recovery by reinforcing adaptive neural plasticity (Jia, 2022). However, while the ERD and ERS changes during MI have been widely studied, there is still limited exploration of how these changes evolve over the course of rehabilitation and their relationship with motor function recovery. Further investigation into the dynamic shifts of ERD and ERS during stroke rehabilitation and their connection to upper limb recovery could provide more personalized rehabilitation strategies for patients and optimize the application of BCI systems in rehabilitation. Some studies suggest that as rehabilitation progresses, EEG signals of stroke patients during MI tasks show signs of improvement, with more pronounced ERD/ERS changes, indicating a recovery of neural function (Bartur et al., 2019; Fong et al., 2021; Kaiser et al., 2012). However, other research indicates that although motor function gradually recovers, the patterns of synchronization and desynchronization do not fully return to normal levels, and this recovery process is often individualized with significant variation across patients (Kancheva et al., 2023; Ray et al., 2020). These findings suggest that ERD and ERS in stroke rehabilitation are influenced not only by individual differences but also by factors such as the specific rehabilitation protocol, the intensity of training, and the neural plasticity (Schranz et al., 2022).

Despite the progress made in understanding ERD and ERS changes during MI tasks, their dynamic evolution during stroke rehabilitation and their relationship with upper limb motor recovery still require further exploration. The present pilot study aimed to evaluate the feasibility and tolerability of MI-based BCI training in stroke rehabilitation and to explore ERD and ERS candidate neural markers of motor imagery–related cortical engagement. Rather than assessing efficacy in a large cohort, this investigation focused on characterizing neural patterns and preliminary functional changes in a small sample, providing proof-of-concept data to inform the design of future controlled trials with extended protocols and systematic follow-up. This work seeks to advance BCI-assisted rehabilitation approaches and provide stroke patients with more optimized rehabilitation strategies.

## 2 Methods

### 2.1 Participants

This is a preliminary study, included three ischemic stroke patients who were recruited from the Department of Rehabilitation Medicine at Shenzhen Longhua District Central Hospital. Inclusion criteria: (1) Diagnosis of ischemic stroke confirmed by neuroimaging (CT or MRI); (2) Stroke onset is the first occurrence, with a stable clinical condition, disease duration between 1 and 48 months, and the presence of upper limb motor dysfunction; (3) Brunnstrom recovery stage ≤ 4 for upper limb and hand function; (4) Modified Ashworth Scale score ≤ 3; (5) No significant cognitive impairment, as determined by a Mini-Mental State Examination (MMSE) score ≥ 18, with the ability to understand instructions and cooperate with therapy (Monroe and Carter, 2012; Tombaugh and McIntyre, 1992); (6) Participants aged between 18 and 80 years, regardless of gender. Exclusion criteria: (1) Presence of other neurological disorders; (2) Acute deterioration, new ischemic stroke, or intracranial hemorrhage during the study; (3) Presence of sensory or mixed aphasia; (4) Participants with a history of epilepsy; (5) Conditions affecting motor function such as fractures or diseases of the affected limb; (6) Patients with significant skull defects or other factors affecting EEG signal collection; (7) Participation in other clinical central nervous system intervention treatments. Drop-out criteria: (1) Participants experiencing significant health decline or adverse reactions during the study; (2) Participants unable to complete follow-up assessments or rehabilitation training; (3) Missing data that affects the evaluation of efficacy or safety; (4) Participants who actively request to withdraw their informed consent during the study. The patients were hospitalized and underwent daily or alternating-day rehabilitation interventions during their stay. See Table 1 for the demographic characteristics of the patients (S01-S03). Clinical characterization of neurological deficits is summarized as follows: S01: Conscious; mild right facial weakness; right Babinski (+); meningismus (−); right upper limb (UL) strength proximal grade 2, distal grade 1; right lower limb (LL) grade 4; left limbs normal strength and tone; Brunnstrom: right UL IV, hand II, LL IV. S02: Conscious; mild right facial weakness; bilateral Babinski (+); meningismus (−); right UL proximal 2+, distal 3; right LL 2+; decreased tone on right; left UL/LL strength 4+, tone normal; Brunnstrom: right UL III, hand I, LL III. S03: Conscious; facial symmetry; Babinski (−); meningismus (−); left UL proximal/distal 4–, left LL 4+; right UL/LL strength 5; tone normal; Brunnstrom: left UL IV, hand II, LL IV. The study was approved by the ethical committee of the Shenzhen Longhua District Central Hospital (Approval No. 2021–001-02) and conducted in accordance with the Declaration of Helsinki. All participants signed informed consent.

**Table 1:** Demographic characteristics of the subjects.

Subjects	Age (year)	Gender	Time post-stroke (month)	Affected body side
01	45	Male	7	Right
02	55	Male	3	Right
03	52	Female	2	Left

### 2.2 Motor imagery training protocol

The BCI training was conducted using the RxHEAL BCI Hand Rehabilitation Training System (Shenzhen RxHEAL Medical Technology Co., Ltd., China). Before the training, patients were instructed to sit upright at the treatment table and were reminded to minimize any movements of the trunk and limbs during the training, except for those required by the protocol.

**Figure 1:**
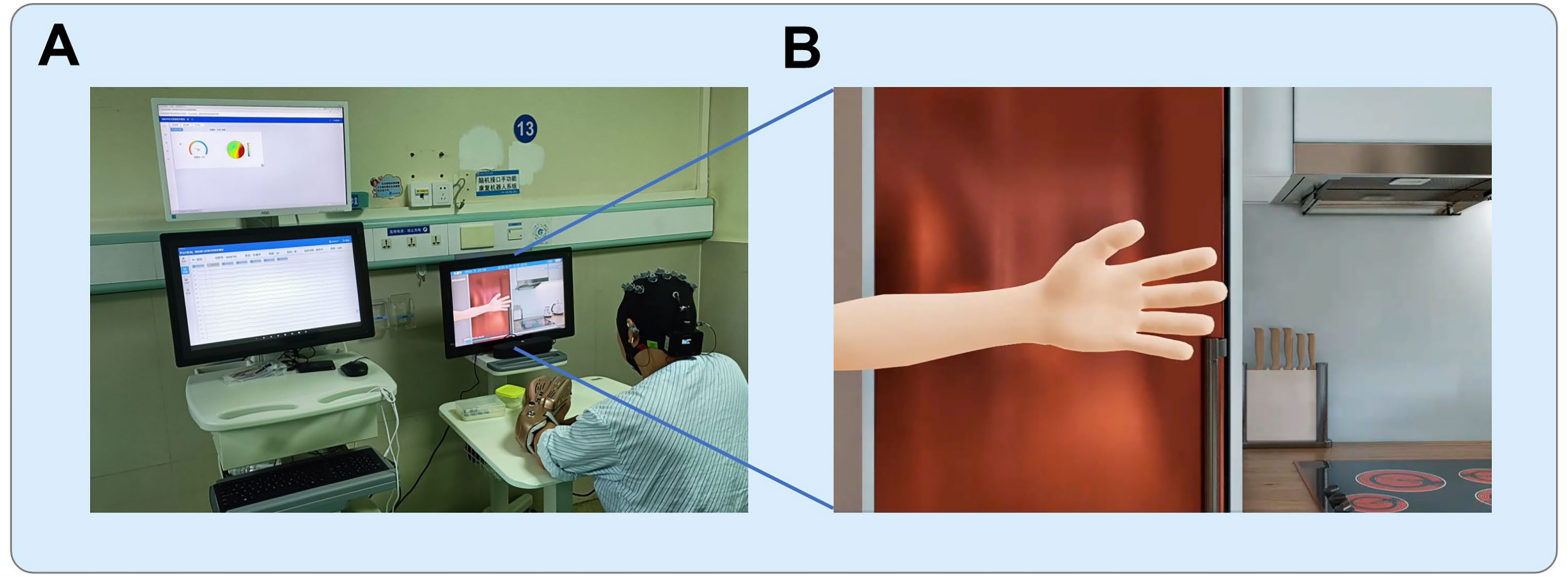
BCI training setup. **(A)** The training environment, with the participant seated and connected to the BCI system, including the EEG cap and robotic exoskeleton. **(B)** The animated scenario displayed on the screen during the training. (© RxHEAL. Reprinted with permission). The animation provides instructions to the patient, prompting them to imagine hand movements, such as grasping or opening, while the system monitors the brain activity and assists with exoskeleton-controlled movements based on the EEG signals.

The BCI rehabilitation system was designed to operate in a closed-loop manner by integrating EEG decoding with multisensory feedback. The exoskeleton robotic hand was fitted onto the affected hand of patient, facilitating MI training. The system allowed multiple movement patterns to be programmed for training. In the present study, two fundamental actions were implemented: whole-hand grasping and whole-hand opening. During each training session, the system software presented auditory instructions and action videos to guide patients in performing MI of the affected hand (Figure 1). EEG signals were continuously recorded during these periods and processed in real time. When the extracted features matched the EEG characteristics associated with MI, the system classified the trial as successful. In such cases, the EEG output was converted into control commands that activated the robotic hand, which executed the corresponding movement and provided tactile feedback in addition to the ongoing auditory and visual cues. Conversely, when EEG features did not meet the MI criteria, no robotic movement was triggered, and feedback indicated an unsuccessful attempt.

These tasks were designed to engage the motor cortex and activate brain regions involved in motor planning and control. Throughout the training, the patient was required to maintain high levels of attention to complete the MI tasks. If the patient made consecutive errors in MI three times, the system would automatically reduce the task difficulty. The training duration was determined by the individual conditions of patient, with most patients completing 10 sets of training in approximately 50 min. Each set consisted of 10 tasks, with 10 sets per session, one session per day, 5 days a week, for 4 weeks. The MI training generally followed this protocol, with appropriate adjustments made based on the individual conditions of the participants. All participants in this study completed at least 15 training sessions.

### 2.3 Concurrent conventional rehabilitation

In addition to the MI training, patients received conventional rehabilitation therapies, including physical therapy, occupational therapy, and speech therapy (if necessary). The conventional rehabilitation aimed to improve the functional recovery, and the MI sessions were intended to complement and enhance these therapies. The combination of interventions allowed for a holistic rehabilitation approach, integrating both motor and cognitive aspects of recovery.

### 2.4 Outcome measurement

The evaluations of motor function were conducted before and after the whole training sessions using the Modified Barthel Index (MBI) (Ohura et al., 2017), the Action Research Arm Test (ARAT) (Yozbatiran et al., 2008), the Fugl-Meyer Assessment Upper Extremity (FMA-UE) (Woodbury et al., 2008), and the Wolf Motor Function Test (WMFT) (Wolf et al., 2001) for all participants. All assessments were carried out by therapists who were blinded to the investigation.

### 2.5 EEG recording and preprocessing

EEG signals were collected throughout the MI training sessions using a 16-channel EEG cap (GREENTEK), with a focus on the sensorimotor cortex (C3, C4, Cz, and surrounding regions), and with A1/A2 used as the reference electrode. Data were sampled at 256 Hz and filtered within a frequency range of 1–100 Hz, with a notch filter applied at 50 Hz to eliminate power line noise. Preprocessing was performed using the FieldTrip (Oostenveld et al., 2011) and EEGLAB (Delorme and Makeig, 2004) toolboxes in Matlab, alongside customized scripts. Artifact detection was first by visual inspection to remove segments with obvious artifacts. Independent component analysis (ICA) was then subsequently applied (Lee et al., 1999), and components were classified using the ICLabel plugin in EEGLAB. Automatic rejection was performed with the pop_icflag function using the threshold array [0 0, 0.9 1, 0.9 1, 0.9 1, 0.9 1; 0.9 1; 0.9 1], corresponding to flagging components in the Muscle, Eye, Heart, Line Noise, Channel Noise, and Other categories if their classification confidence exceeded 90%. Under these criteria, no components met the rejection threshold, and thus no trials were excluded at this stage. A likely explanation is that overt artifacts (e.g., large blinks or muscle bursts) had already been minimized during the initial visual rejection and that the MI training sessions were conducted under controlled conditions with participants instructed to minimize extraneous movements. The data were then segmented into 4-s epochs, starting 4 s before the trial offset and ending at the trial offset.

### 2.6 Data analysis / statistics

Time-frequency analysis was conducted within a frequency range of 3–30 Hz, focusing specifically on ERD and ERS to track dynamic neural responses during the MI tasks. The spectral analysis across the entire electrode array was performed using the Hanning taper method with a 5-cycle time window, applying frequency smoothing with 1 Hz steps and variable width depending on the frequency. For each epoch, the analysis window spanned from 3 s before the trial offset to 1 s before the offset. Baseline correction was performed using a 2-s pre-onset period, and the data were expressed in decibels (dB). The EEG signatures of frequency and power from the C3 and C4 electrodes during MI were derived from the average of all training sessions for each participant. To compare the differences between the C3 and C4 electrodes, the Wilcoxon signed-rank test was conducted for each frequency bin at the training-session level, with significance set at *p* < 0.05. Additionally, regression analysis was employed to examine any trends in changes of neural activities over the course of training, assessing whether the power within the specified frequency bands showed significant increases or decreases as a result of the training.

## 3 Results

**Figure 2:**
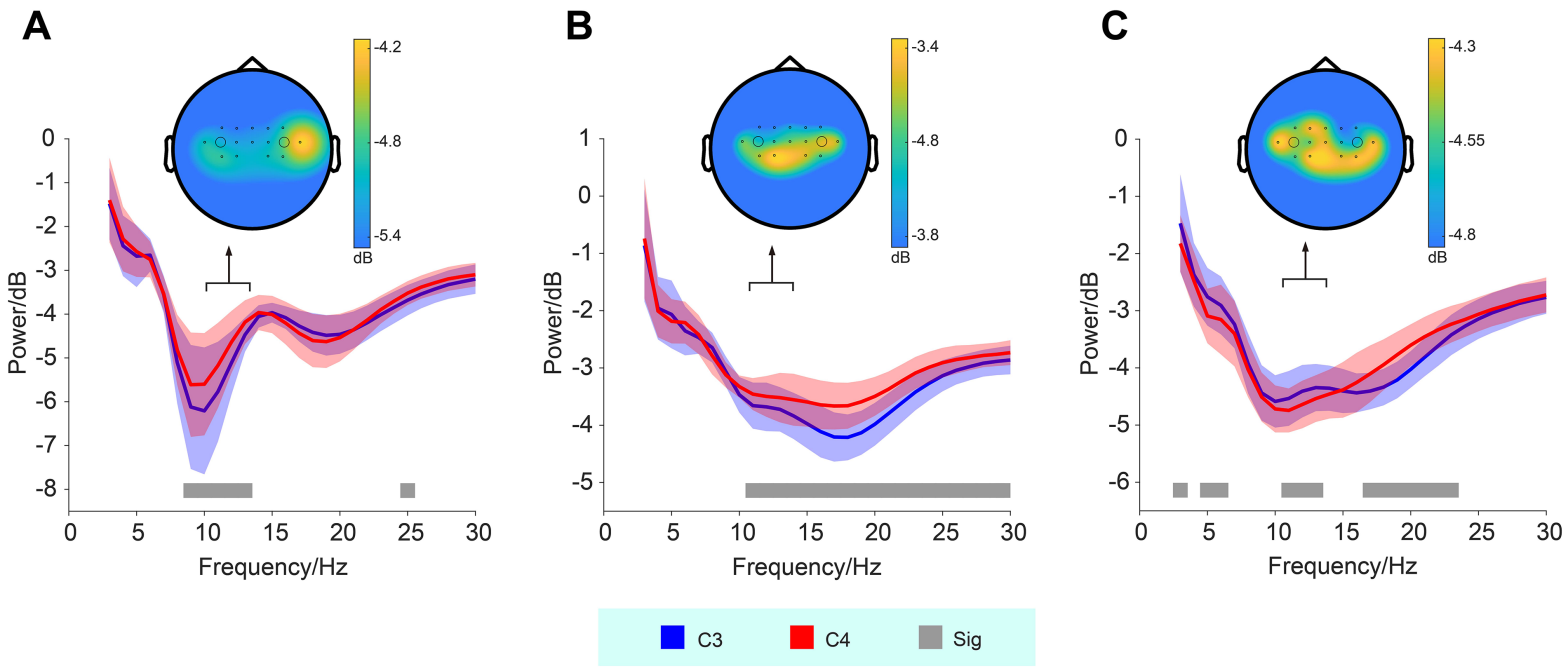
Frequency and power of EEG signals recorded from the C3 and C4 electrodes during MI. Panels **(A–C)** represent subjects 01–03, respectively. Significant differences (Sig) in high-alpha band activity between the C3 and C4 electrodes are observed across all three subjects, indicated by the gray bars (Wilcoxon signed-rank test, *p* < 0.05). The shaded areas represent the standard deviation. Corresponding topographic maps of high-alpha band activity are shown, with black dots indicating the electrode locations, and larger circles denoting the positions of the C3 (left) and C4 (right) electrodes.

The power of EEG frequency (3–30 Hz) recorded from the C3 and C4 electrodes during training sessions was analyzed in three subjects. The corresponding spectral plots (averaged across training sessions) for each subject are presented in Figure 2. For all three subjects, significant differences in the power of high-alpha activity between the C3 and C4 electrodes were observed, particularly in the frequency range around 11–13 Hz (Wilcoxon signed-rank test, *p* < 0.05). For Subject 01, the mean power (dB) was −5.12 ± 0.73 (means ± std) at C3 and −4.66 ± 0.71 at C4. Subject 02 exhibited mean powers of −3.69 ± 0.39 at C3 and −3.49 ± 0.35 at C4, while for Subject 03, the values were −4.43 ± 0.43 at C3 and −4.64 ± 0.38 at C4. In Subject 02, we observed a consistent trend between the beta band and high-alpha band activity, with both showing ERD at the C3 location compared to C4. In contrast, in Subject 03, a reverse trend was found: beta band activity exhibited more ERD at C3, while high-alpha band activity showed more ERD at C4, suggesting differential modulation of these frequency bands across the electrodes.

In addition to the spectral plots, topographic maps (see Figure 2) illustrate the spatial distribution of high-alpha activity. These maps demonstrate marked bilateral hemispheric asymmetry in the spatial distribution of the high-alpha band across subjects.

**Figure 3:**
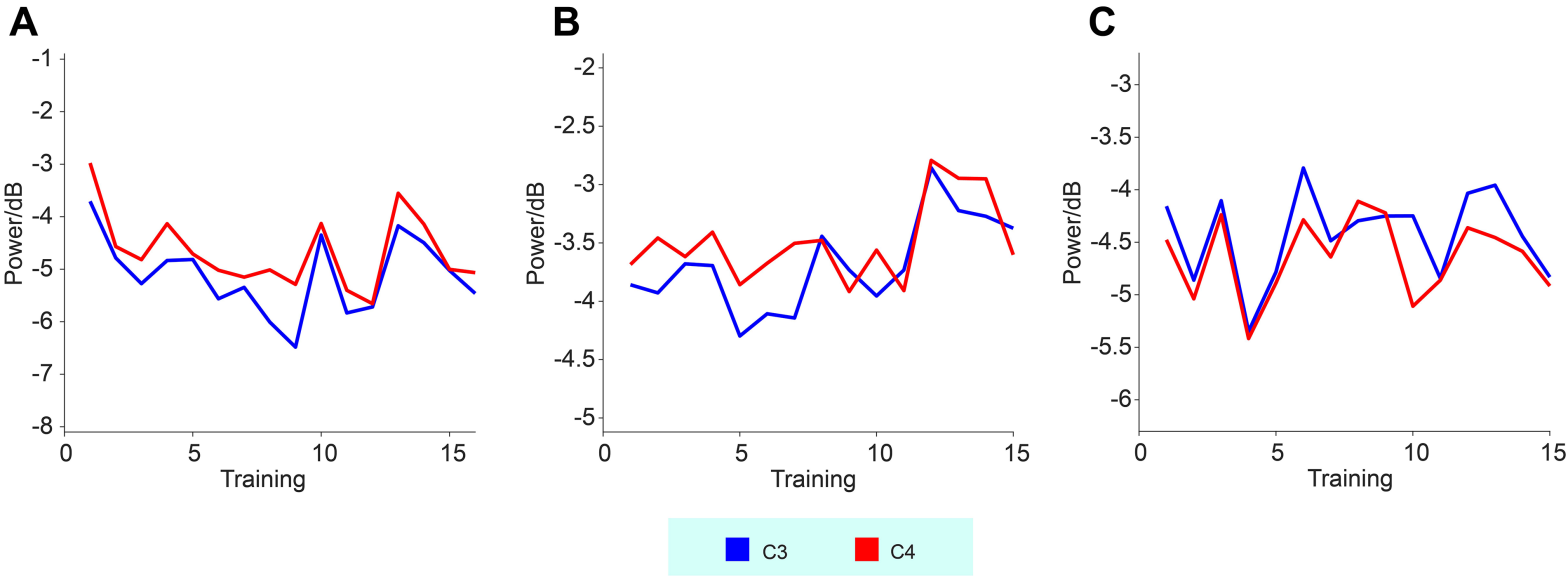
High-alpha band EEG activities recorded from the C3 and C4 electrodes across three subjects. Panels **(A–C)** represent subjects 01–03, respectively. The curves show the changes in high-alpha power as a function of training sessions.

The changes in high-alpha band activity as a function of training sessions were also analyzed, as presented in Figure 3. However, regression analysis did not reveal any significant trends or changes in the high-alpha band power across sessions.

Upper limb function assessments were conducted before and after training, and the results are presented in Table 2. It can be observed that all three subjects showed improvements in upper limb function throughout the entire process.

**Table 2:** Upper limb function scores.

Subjects	MBI			ARAT			FMA-UE			WMFT		
	Pre	Post	Δ	Pre	Post	Δ	Pre	Post	Δ	Pre	Post	Δ
01	100	100	0	13	24	+11	21	37	+16	28	38	+10
02	20	30	+10	1	6	+5	2	11	+9	0	10	+10
03	76	100	+24	5	34	+29	8	53	+45	8	45	+37

MBI, Modified Barthel Index; ARAT, Action Research Arm Test; FMA-UE, Fugl-Meyer Assessment Upper Extremity; WMFT, Wolf Motor Function Test; Pre, pre-training; Post, post-training; Δ, Post–Pre.

## 4 Discussion

The present study investigated how ERD and ERS patterns during MI tasks are involved in the rehabilitation process of ischemic stroke patients, specifically those undergoing MI-based BCI training with robotic hand assistance. Our EEG analysis revealed a consistent trend in the ERD of the high-alpha band and individual differences in the neural activity across participants. In addition, all three participants showed improvements in upper limb function throughout the training period, as indicated by functional assessments. These findings suggest that MI-based BCI training may have a beneficial effect on motor recovery in ischemic stroke patients, supporting previous research that highlights the potential of BCI systems for rehabilitation.

EEG analysis revealed significant differences in high-alpha band power between the C3 and C4 electrodes. Stronger ERD was observed in the contralesional side of the hemiparetic patients. For example, Subject 01 and 02 showed stronger high-alpha ERD at C3, while Subject 03 displayed stronger high-alpha ERD at C4. The topographic maps further revealed electrode-specific differences in high-alpha band activity, underscoring individual variations in neural modulation during MI tasks. These findings are consistent with prior studies indicating that MI, particularly in stroke rehabilitation, is associated with specific patterns of cortical activation, which may be localized to the sensorimotor areas corresponding to the affected limb (Chen et al., 2022; Shahid et al., 2010).

Furthermore, the relationship between high-alpha and beta band activity was explored. In Subject 02, a consistent trend was observed between both frequency bands, with C3 showing greater ERD than C4. This suggests a potential coupling of neural activity in both bands during MI, which could reflect enhanced cortical engagement during motor preparation or execution (Gerloff et al., 2006). However, in Subject 03, the beta band and high-alpha band activity exhibited opposing trends. Specifically, C3 showed greater ERD in the beta band, while C4 exhibited more ERD in the high-alpha band. This reversal in neural activity patterns may reflect individual differences in how stroke patients engage neural resources during MI tasks and could provide insight into personalized approaches to BCI-based rehabilitation (Kancheva et al., 2023).

Regression analysis of the changes in signal intensity over the course of the training sessions did not reveal significant trends in either the high-alpha band, suggesting that the high-alpha band neural activity during MI remained relatively stable throughout the training. This result indicates that MI-based BCI training may require longer durations or more intensive sessions to produce measurable changes in neural activity (Rimbert and Fleck, 2023). It is also possible that the lack of significant changes reflects the individualized nature of stroke recovery, where improvements in motor function may not always correlate with immediate changes in neural activity.

The overall improvement in upper limb function observed across all subjects suggests that MI-based BCI training can potentially facilitate motor recovery in stroke patients by engaging residual neural resources (Curado et al., 2015). Contralesional high-alpha ERD may reflect compensatory recruitment of motor circuits after stroke, and recent evidence further indicates that MI-BCI training can enhance EEG-based functional connectivity in motor networks, with such changes correlating with clinical improvements (Kim et al., 2025). Although the EEG changes observed here were subtle, they align with the growing body of evidence supporting the role of MI in modulating cortical activity and enhancing motor rehabilitation (Ma et al., 2024; Monteiro et al., 2021). Further studies with larger sample sizes and longer training periods are needed to elucidate the precise mechanisms underlying the neural changes associated with MI-based BCI training and to determine how these changes relate to functional improvements in stroke rehabilitation.

While the findings of this study provide promising insights into the potential of MI-based BCI training for stroke rehabilitation, several limitations must be considered. First, the study included only three participants, which substantially limits generalizability. These findings should therefore be interpreted as preliminary, and larger samples are needed to draw broader conclusions. Moreover, this study was intentionally designed as a pilot case-series study focusing on feasibility, tolerability, and the exploration of neural markers in a real-world clinical setting. Although our small sample did not allow for a direct statistical association between high-alpha ERD and functional improvements, highlighting this potential mechanistic linkage is important for guiding future research. Accordingly, future studies should incorporate randomized or sham-controlled designs to distinguish BCI-specific gains from conventional rehabilitation or recovery and to strengthen causal inference and generalizability. Finally, the duration of the training sessions may have been insufficient to reveal significant trends in neural activity; longer training periods may provide more robust evidence of training-induced neuroplasticity.

In conclusion, this study demonstrates the potential of MI-based BCI systems for upper limb rehabilitation in stroke patients, with a particular focus on the role of ERD in monitoring neural activity during rehabilitation. Our findings suggest that changes in the ERD of the high-alpha band reflect neural reorganization associated with motor recovery. The individual differences observed in neural responses highlight the importance of personalizing BCI training protocols to optimize rehabilitation outcomes. Future work should explore the long-term effects of BCI training on both neural activity and motor function, as well as the potential for integrating MI-based BCI with other neuroplasticity-promoting therapies.

## Data Availability

The data are not publicly available due to privacy and ethical considerations. The data supporting this study are available upon request from the corresponding author.
